# A Case of Aortic Dissection Presenting with Atypical Symptoms and Diagnosed with Transthoracic Echocardiography

**DOI:** 10.1155/2019/6545472

**Published:** 2019-11-11

**Authors:** Mahdis Solhjoo, Supreeya Swarup, Amgad N. Makaryus

**Affiliations:** ^1^Departments of Medicine, Nassau University Medical Center, East Meadow, NY, USA; ^2^Department of Cardiology, Nassau University Medical Center, East Meadow, NY, USA; ^3^Zucker School of Medicine at Hofstra/Northwell Health, Hempstead, NY, USA

## Abstract

We present a case of an extensive aortic dissection (AD) identified in a woman with atypical symptoms. Transthoracic echocardiography (TTE) allowed the identification of an intimal flap in multiple locations and resulted in rapid diagnosis and treatment. In most cases, CT angiography is the imaging modality of choice for diagnosis of AD. TTE is rapid and accurate and can be used in kidney failure. Our case highlights the important role of bedside echocardiography in the diagnosis of AD, especially in the patient with a typical symptoms in whom this diagnosis of AD may not be entertained and actually missed leading to negative and possibly deadly consequences.

## 1. Introduction

The diagnosis of aortic dissection (AD) can be challenging given an extensive range of symptoms at presentation. The mortality rate for untreated patients with AD after symptoms start is about 1% per hour. As a result, early diagnosis is required for proceeding with the most appropriate medical or surgical treatment options [1]. In one study, patients with AD had been misdiagnosed in 16% of cases presenting to the ER and AD was not even included in the initial differential diagnoses [2].

One of the fastest and most cost-effective diagnostic methods for AD is the bedside echocardiogram. We present a case of AD and highlight the important role of bedside echocardiography in the diagnosis, especially in the patient with atypical symptoms in whom this diagnosis of AD may not be entertained and actually missed leading to negative and possibly deadly consequences.

## 2. Case Summary

A 48-year-old woman presented to the emergency department (ED) with complaints of dizziness, generalized weakness, nausea and diarrhea. The patient assumed her blood pressure was elevated, however, despite oral medications, her symptoms persisted prompting her presentation to the ED. She denied any chest pain, back pain, headache and changes in the vision. She stated that she felt weak on her left side and was unable to move her left lower extremity. The patient's medical history consisted of hypertension, hyperparathyroidism and stage 3 chronic kidney disease. Her home medications for hypertension included Losartan, Amlodipine, Hydralazine and, Carvedilol.

**Figure 1:**
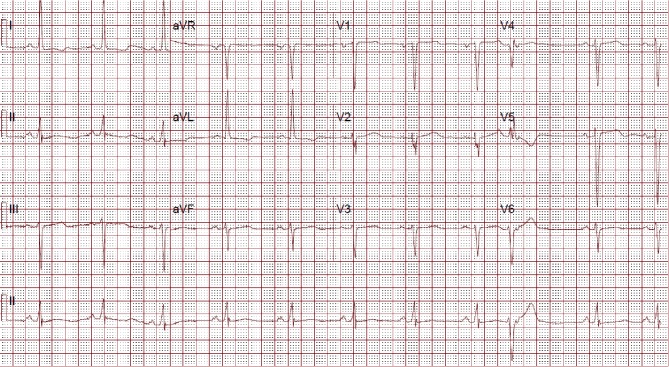
EKG showing occasional ventricular premature complexes, left ventricular hypertrophy, with nonspecific ST-segment, *T*-wave changes.

In the ED, the initial vital signs were blood pressure of 125/85 mmHg in the right arm and 119/83 mmHg in the left arm, pulse rate 64/min, respiration rate 18/min, temperature 97.4°F and oxygen saturation of 99% on room air. The patient was in no acute distress. The cardiac exam showed a normal S1 and S2 with no murmur and the peripheral exam showed normal bilateral peripheral pulses. The neurologic exam showed cranial nerves 2–12 intact with grossly normal motor strength. Electrocardiogram (EKG) showed sinus rate at 63 bpm with occasional ventricular premature complexes, left ventricular hypertrophy and nonspecific ST-segment, *T*-wave changes (Figure 1). Laboratory findings were as follows: creatine kinase (CK) 139s IU/L, CK-myocardial band 2.8 IU/L, troponin I 0.34 ng/mL, white blood cell 15480/*μ*L, hemoglobin 14.6 g/dL, creatinine 2.9 mg/dl and eGFR 17 ml/min/1.73 m^2^.

**Figure 2:**
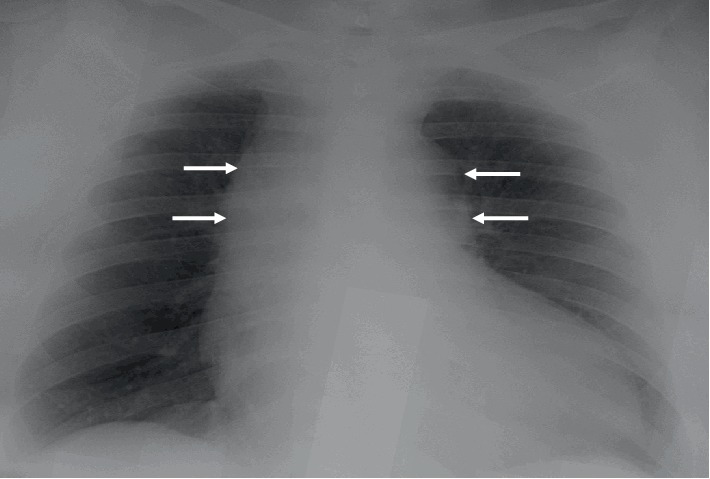
CXR showing widened mediastinum (arrows), enlarged cardiomediastinal silhouette and longitudinal retrocardiac opacity.

**Figure 3:**
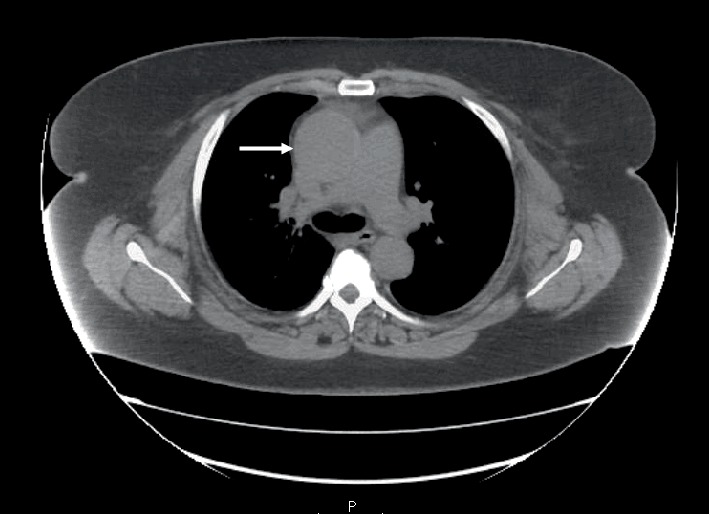
Axial CT image of the thorax without contrast showing aneurysmal dilatation (arrow) of the ascending thoracic aorta.

**Figure 4:**
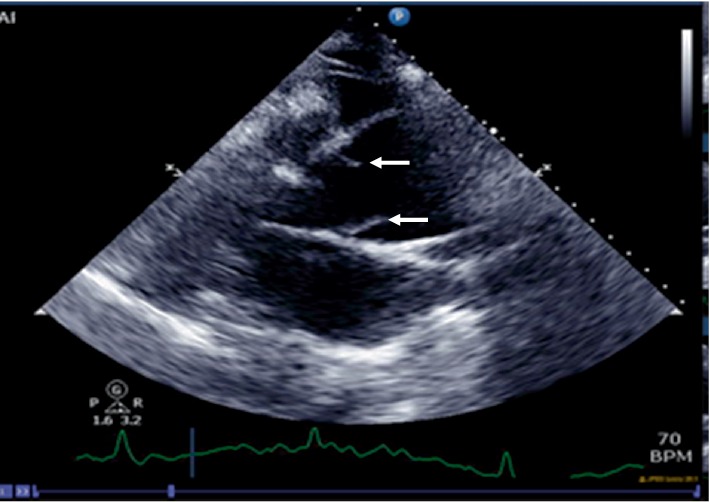
Parasternal long axis view showing the dissection flap (arrows) in the ascending aorta.

**Figure 5:**
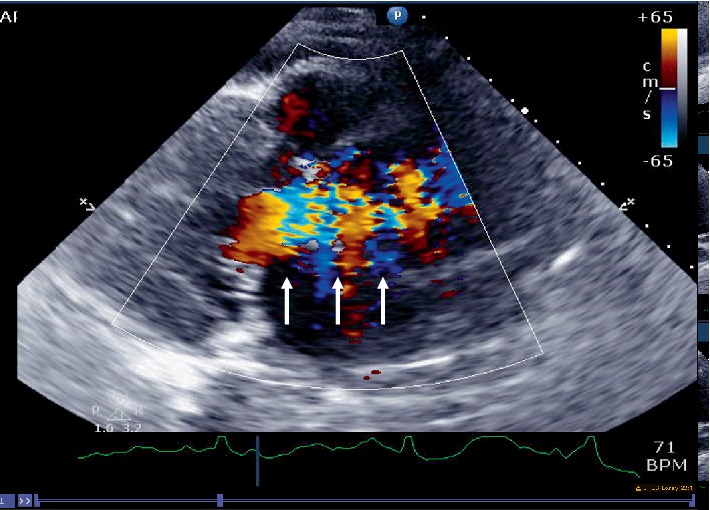
Parasternal long axis view showing aortic insufficiency (arrows).

**Figure 6:**
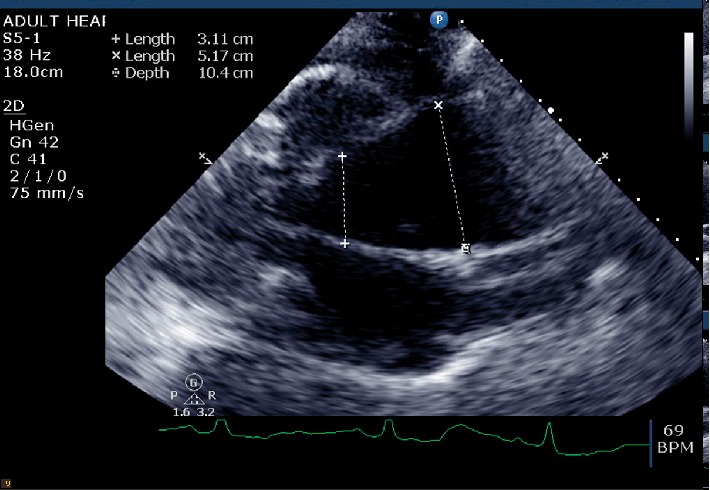
Parasternal long axis view showing the ascending aortic aneurysm.

**Figure 7:**
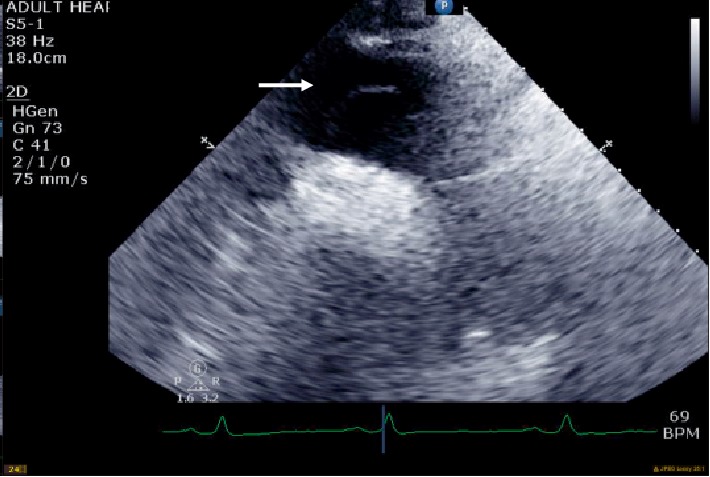
Suprasternal notch view showing the dissection flap extending to the aortic arch (arrow).

**Figure 8:**
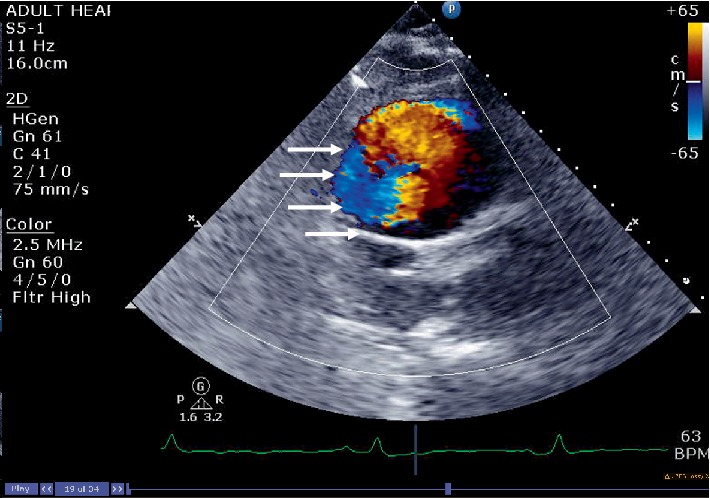
Color Doppler helps in appreciating the true lumen (arrows) from the false lumen.

**Figure 9:**
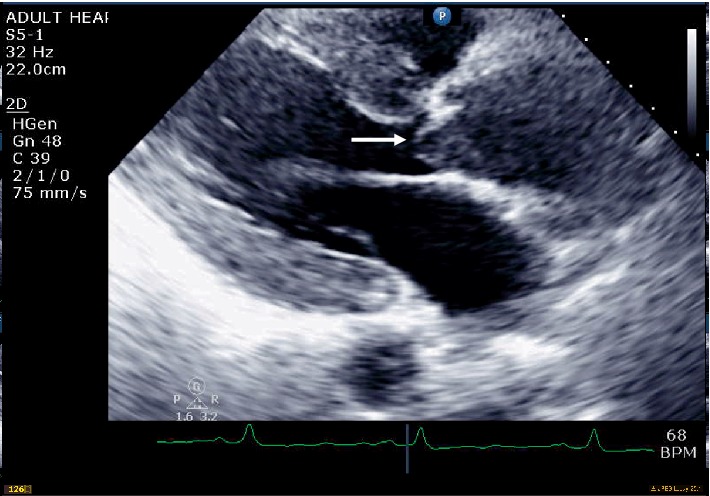
Parasternal long axis view showing dissection flap prolapsing (arrow) into the left ventricular outflow tract causing aortic insufficiency.

The chest X-ray (CXR) showed a widened mediastinum, enlarged cardiomediastinal silhouette, and longitudinal retrocardiac opacity which was noted by the radiologist to possibly reflect a thoracic aortic aneurysm (Figure 2). Because the patient had elevated creatinine, computed tomography (CT) of thorax without contrast was performed. The CT showed aneurysmal dilatation of the ascending thoracic aorta, measuring up to 4.5 cm in the axial image (Figure 3). From the data above (aortic dissection detection risk score of 1), and given the fact that the Troponin I levels were uptrending, (1.340 ng/mL–1.430 ng/mL), evaluation for dissection was undertaken initially utilizing a bedside transthoracic echocardiogram (TTE) which identified an ascending aortic/aortic root aneurysm with a dissection flap (Figures 4–9). Elevating troponin with ascending aortic aneurysm and flap seen on TTE was consistent with type A aortic dissection. The patient was sent for urgent cardiothoracic surgery undergoing transesophageal echocardiography intraoperatively to fully elucidate the dissection extent and underwent a composite graft replacement of the aortic valve, aortic root, and ascending aorta, with re-implantation of the coronary arteries into the graft (Bentall procedure). The patient did well post-surgery without complication.

## 3. Discussion

Chest pain is one of the most common symptoms in dissection and its absence makes the diagnosis difficult for the clinician. One study found that about 71% of patients with type A dissections presented with anterior chest pain while 6% did not have any pain [3]. The most common initial diagnosis in patients with AD are acute coronary syndrome, cerebrovascular accidents, and gastrointestinal and pulmonary diseases. Absence of chest pain, presentation of neurologic deficits, syncope, vascular insufficiency and gastrointestinal (GI) symptoms are some of the symptoms leading to misdiagnosis of AD [3–5].

Women are also at increased risk of AD misdiagnosis since AD is less common in women, usually presents in older women, and has more atypical symptoms [6, 7]. Less than 5% of patients with AD present with GI manifestations such as nausea, vomiting, and bloody diarrhea [8]. In one case report, a patient presented with severe diarrhea and was finally diagnosed with AD [9]. It is therefore important to have a high clinical suspicion of AD in patients with risk factors and atypical symptoms.

In our case, the patient has multiple factors that routinely lead to misdiagnosis. Our case is a young female with GI symptoms and no specific chest pain. Being aware of having hypertension and noting an aortic aneurysm can be helpful to put the AD in the initial differential diagnoses list. Although the CXR can be used as a screening tool in chest pain, only about 50% of patients with AD have a widened mediastinum or abnormal aortic contour. Studies have found CXR sensitivity and specificity for AD of only 67% and 70%, respectively [3, 10]. Troponin levels are positive in about 50% of the patients with AD [11]. Also, EKG abnormalities can present in AD similar to myocardial infarction patients. Most common EKG findings are the presence of nonspecific ST-segment or *T*-wave changes and rarely ST-segment elevation or new *Q* waves [12]. Both high troponin levels and EKG changes can cause confusion and delay in AD diagnosis that leads to inappropriate treatment with antithrombotics which can cause more complications [13].

Preferred imaging studies in the diagnosis of AD are echocardiography and CT in the emergency setting and magnetic resonance (MRI) for the stable patients. Although CT and MRI give a better field of view, the role of the echocardiography is very important since it is rapid, portable, and cost-effective in the diagnosis. TTE can assess different aortic segments and visualize an intimal flap in the proximal ascending aorta. In past, the role of TTE was limited in the diagnosis of AD because its sensitivity was very low in the diagnosis of descending AD (31–55%). Currently, with the newest imaging technology, TTE sensitivity and specificity have been improved in the diagnosis of AD [14]. Although trans-esophageal echocardiography (TEE) is the better technique, in many studies TTE has been suggested as the initial imaging in the emergency setting because of its availability and rapidity in the diagnosis of AD [11, 15, 16]. In our case, AD was diagnosed by TTE. This case emphasizes the role of clinical suspicion of AD. Furthermore, many patients with typical and atypical symptoms for ascending AD can be easily diagnosed with bedside TTE that is both fast and accurate. Recent studies are discussing the importance of TTE in early diagnosis of AD which can be lifesaving mainly in patients with atypical symptoms.
